# Hospitalization and Health Resource Utilization in Emergency Department Cases of Diabetic Foot Infections in the U.S. from 2012 to 2021: A Nationally Representative Analysis

**DOI:** 10.3390/jcm13185361

**Published:** 2024-09-10

**Authors:** Matthew C. Dickson, Grant H. Skrepnek

**Affiliations:** College of Pharmacy, The University of Oklahoma Health Sciences, Oklahoma City, OK 73117, USA; matthew-dickson@ouhsc.edu

**Keywords:** diabetic foot infection, diabetic foot, diabetes complications, diabetes, hospitalization, length of stay, nationally representative

## Abstract

**Objectives:** The objective of this paper was to assess hospitalizations and health resource utilization associated with diabetic foot infection (DFI)-related visits within emergency departments (EDs) in the U.S. **Methods:** This nationally representative, cross-sectional historical cohort utilized the Centers for Disease Control and Prevention’s (CDC’s) National Hospital Ambulatory Medical Care Survey across a ten-year period from 2012 to 2021. Inclusion criteria were as follows: adults ≥18 years of age; a diagnosis of Type 1 or Type 2 diabetes mellitus; presence of a DFI. Comparisons were drawn relative to a cohort of patients with diabetes without foot complications. Study outcomes included 72-hour (72 h) ED revisit, hospitalization, and length of stay (LOS). Top diagnoses and medications were also reported. Multivariable, generalized, linear regression analyses were employed, controlling for key demographics, health system factors, clinical characteristics, and year. **Results:** An estimated 150.6 million ED visits included a diabetes diagnosis, with 2.4 million involving a DFI (1.6%). Approximately half of DFI cases were hospitalized (43.7%). Anti-infective medications were prescribed in 83.1% of DFI cases, including vancomycin in 28.1%. Multivariable analyses observed that DFIs were associated with a 3.002 times higher odds of hospital admissions (CI: 2.145–4.203, *p* < 0.001) and a 55.0% longer LOS (IR = 1.550, CI: 1.241–1.936, *p* < 0.001). DFIs were not significantly associated with a 72 h ED revisit. **Conclusions:** This nationally representative study of 2.4 million DFI-related ED visits in the U.S. observed higher odds of hospital admissions and a longer LOS for DFIs versus diabetes without foot complications. Continued research should seek to assess prevention and coordinated treatment interventions prior to the emergence of DFIs requiring ED care.

## 1. Introduction

Diabetic foot ulcers (DFUs) and diabetic foot infections (DFIs) are severe complications of diabetes and are principally caused by neuropathy and ischemia [1]. An estimated 38.4 million persons (11.6%) of the U.S. population were living with diabetes in 2021 and, globally, an estimated 529 million (6.1%) people were living with diabetes [2,3]. Persons with diabetes have a 19–34% lifetime risk of developing a DFU, and almost half of all DFUs progress to a DFI, which results in increased hospitalizations, amputations, and death [4,5,6,7]. Diabetic foot complications are the leading cause of amputations in diabetes and in amputations overall, and have been estimated to account for one-third of all cost of diabetes, where the economic cost of diabetes in the U.S. was estimated at USD 412.9 billion in 2022 (i.e., USD 306.6 billion in direct healthcare expenditures and USD 106.3 billion in lost productivity from work-related absenteeism, reduced productivity at work and at home, unemployment from chronic disability, and premature mortality) [8,9,10].

Regarding the occurrence of DFIs, Duhon et al. (2016) reported that the incidence of DFIs lowered from 2.3% of diabetes-related discharges in 1996 to 1.1% in 2010 [11]. The annual incidence of DFUs has been estimated to range between 0.1 and 8% in community- and population-based cohort studies [5]. Skrepnek et al. (2015) analyzed adult visits to the emergency department (ED) in the U.S. in 2006–2014 and reported that 54.2 million estimated cases involved any diagnosis of diabetes, with 1 million estimated cases involving any diagnosis of a DFI [12]. Approximately 80% of cases were subsequently hospitalized, 10% experienced major/minor amputations, and there the mortality rate was 2% [12]. Several studies have reported that the diabetes-related amputation rate decreased from 1990 to 2010 [12,13,14,15]. However, Geiss et al. (2018) also reported that amputation rates increased from 2010 to 2015, though they were predominately minor amputations [15]. As DFIs frequently require emergency medical care and may lead to hospital admission and surgical intervention, updated national estimates on the national burden of DFIs are warranted. The objective of this study was to assess hospitalizations and health resource utilization associated with DFI-related visits in EDs in the U.S.

## 2. Methods

This nationally representative, cross-sectional historical cohort study utilized the Centers for Disease Control and Prevention’s (CDC’s) National Hospital Ambulatory Medical Care Survey (NHAMCS) data from 2012 to 2021 [16,17]. These data are publicly available, fully de-identified, and sampled from ED visits within non-institutional, non-federal, general, short-stay hospitals [16]. The basic sampling unit is the patient visit or encounter within each year. Thus, patient-level assessments are restricted to a cross-sectional perspective, without the possibility of incorporating longitudinal elements [16]. These data contain elements related to the visit, including information about the patient, treatment, and services; certain outcomes; and ED characteristics [16]. Overall, utilizing NHAMCS data can provide information on the patterns of care and health resource utilization for ED visits.

Inclusion criteria were as follows: adults ≥18 years of age; a diagnosis of Type 1 or Type 2 diabetes mellitus; the presence of a DFI. Comparisons were drawn relative to a cohort of patients with diabetes and without foot complications (i.e., DFUs without infection were excluded from comparison due to changes in ICD-10-CM codes and inherent NHAMCS data restrictions). A DFI diagnosis was determined utilizing a validated algorithm reported by Sohn et al. (2010), utilizing ICD-9-CM diagnosis codes and converted to corresponding ICD-10-CM diagnosis codes [18]. Additionally, a diagnosis of DFI was determined via the CDC’s reason-for-visit codes [19]. To derive reasons for visits pertinent to this study, a manual review of the reason for visit classification codes was conducted and appropriate matches to the validated ICD-9-CM diagnostic codes were utilized. Specifically, the inclusion criteria were any listed reason-for-visit code, including infection (i.e., cellulitis, abscess, osteomyelitis, and gangrene) in combination with any listed reason-for-visit code, including foot-related concerns in a case indicating a diabetes diagnosis. A full listing of ICD-9-CM, ICD-10-CM, and reason-for-visit codes utilized to determine DFIs and diabetes are presented in Appendix A.

This study’s outcomes included 72 h ED revisits, hospitalization, and inpatient length of stay. Baseline descriptives were analyzed to assess the differences between cohorts utilizing a bivariable generalized linear model framework, with proportions or means ± standard deviations reported. The inferential analysis used multivariable, generalized, linear regression models that controlled for key demographics (i.e., age, sex, race, and primary insurance coverage), health system characteristics (i.e., rural/urban physician practices, geographic region, and practitioner level), clinical characteristics (i.e., Deyo–Charlson Comorbidity Index, the Emergency Severity Index, and COVID-19), and year [20,21,22,23,24]. The Deyo–Charlson Comorbidity Index is a measure of case–mix disease severity and has been validated for mortality and resource utilization [21]. The ESI measures the severity of the case presenting to an ED and has been validated in hospitalization and resource utilization [22]. Specifically, binomial/logistic generalized linear models were used to analyze 72 h ED revisit and hospitalization, and a negative binomial generalized linear model was used to analyze an inpatient length of stay [24]. Post hoc sensitivity analyses were conducted to assess controlling for sepsis with DFI and independent of DFI. Additional descriptive analyses were conducted to assess the frequency of diagnosis codes and medications utilized or prescribed in an ED visit. To yield nationally representative estimates, Taylor series methods were incorporated in calculating standard errors [16]. Significance was set at alpha 0.05. All analyses were conducted with Stata MP 16.2 (College Station, TX, USA).

## 3. Results

### 3.1. Descriptive Statistics

**Figure 1:**
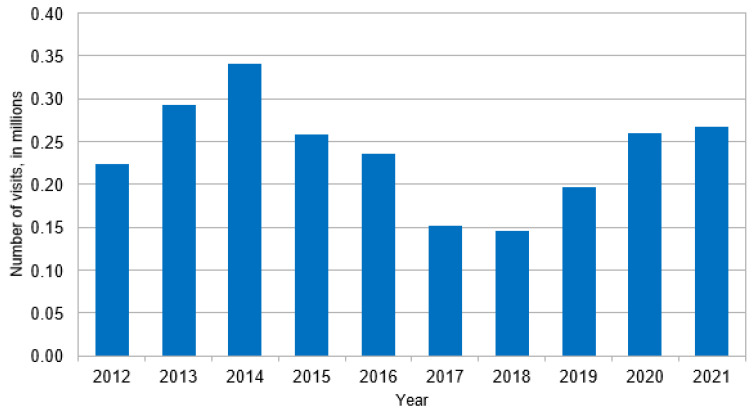
Estimated number of diabetic foot infections by year.

**Figure 2:**
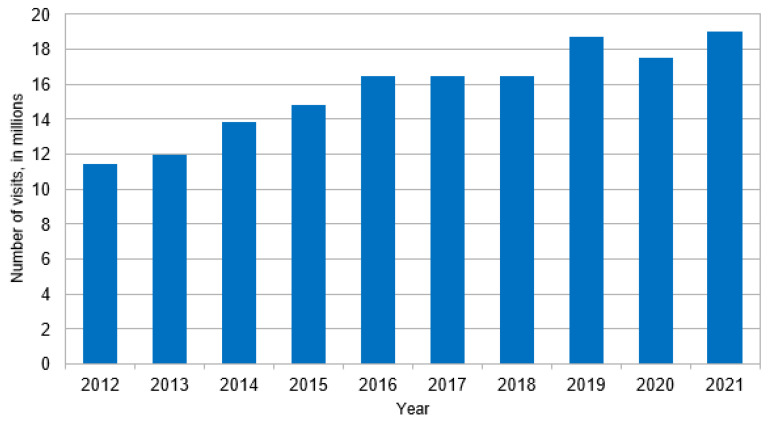
Total cases involving diabetes, including visits with diabetic foot complications.

**Figure 3:**
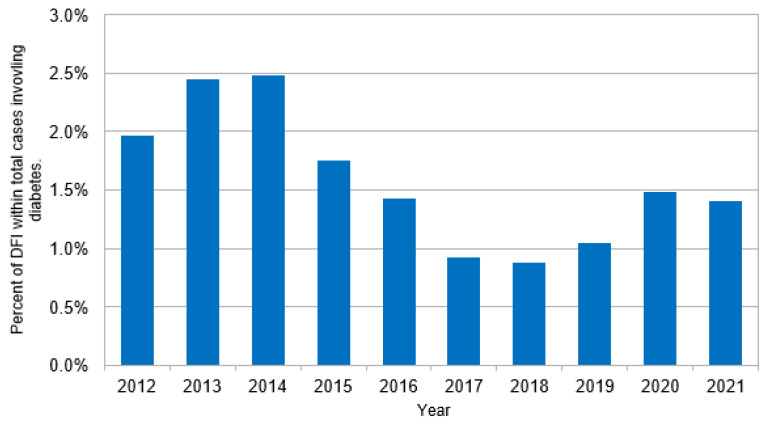
Estimated cases of diabetic foot infections (DFIs) as a percentage of total diabetes cases.

Reported in Table 1, an estimated 153.0 million ED visits in the U.S. from 2012 to 2021 included a diabetes diagnosis, including 2.4 million DFI cases (1.6% of all visits involving diabetes). Compared to diabetes without foot complications, DFI cases were significantly younger (57.4 ± 12.9 years vs. 60.2 ± 14.4, *p* < 0.05), more often male (DFI _Male_ 62.1% vs. diabetes-alone _Male_ 45.2%, *p* < 0.001), and with a higher proportion of white race (DFI _white race_ 80.5% vs. diabetes-alone _white race_ 70.3%, *p* < 0.01). Concerning clinical characteristics, DFI cases had a higher proportion of sepsis (5.7% vs. 1.7%, *p* < 0.001), though the differences in ESI scores indicate that DFI cases were overall less severe. An ESI Level-5 is the best-case scenario and was more common in DFI than in diabetes without foot complications (DFI_ESI Level-5_ 42.7% vs. diabetes-alone _ESI Level-5_ 29.1%, *p* < 0.001). An ESI Level-3 case represent an ‘urgent’ case and was also more common in DFI cases (DFI _ESI Level-3_ 30.4% vs. diabetes-alone _ESI Level-3_ 25.3%, *p* < 0.05). ESI Level-2 cases were more common in diabetes without foot complication cases than DFI cases (DFI _ESI Level-2_ 8.3% vs. diabetes-alone _ESI Level-2_ 32.7%, *p* < 0.001). A greater proportion of the sample of DFI cases were observed in the years 2013 and 2014 than in diabetes without foot complications, whereas DFI had a lower proportion of cases in the years 2017 and 2018. Figure 1, Figure 2 and Figure 3 present the estimated DFI cases per year, the estimated total cases of diabetes, and the proportion of total diabetes cases that involved DFI, respectively. Overall, the average number of DFI cases per year across the ten-year study horizon was 23.7%, though the proportion of DFI relative to diabetes varied over time. In more detail, the percent of total diabetes cases that involved DFIs peaked in 2014 (2.5%), was lowest in 2018 (0.8%), and reached 1.4% in 2021. The estimated number of cases involving diabetes overall increased over time, from 11.4 million in 2012 to 19.0 million in 2021.

Regarding study outcomes, shown in Table 1, the unadjusted results indicated that DFI cases were admitted to hospital significantly more than diabetes without foot complications (DFI _Admit_ 43.7% vs. diabetes-alone _Admit_ 27.3%, *p* < 0.001). Once admitted, DFI cases stayed significantly longer than diabetes without foot complications (DFI _LOS_ 8.4 days ± 5.7 vs. diabetes-alone _LOS_ 5.6 days ± 4.8, *p* < 0.01). Less than ten percent of DFI cases or diabetes without foot complication cases reported being a 72 h ED revisit with no significant difference between the cohorts.

#### Top Medications and Diagnoses

Regarding medications prescribed for DFIs, anti-infective medications were the most frequently prescribed class and present in 83.1% of cases. The most highly utilized anti-infective in DFI cases was vancomycin, with an estimated 28.1% of prescriptions. Post hoc analyses indicated that 31.8% of DFI cases were prescribed an anti-infective agent with Methicillin-resistant *Staphylococcus aureus* (MRSA) (e.g., vancomycin) or *Pseudomonas aeruginosa* (e.g., piperacillin-tazobactam) coverage. ‘Cellulitis or acute lymphangitis of other parts of limb’ was the most common diagnosis in DFI cases, with an estimated 37.3% of observations. The top ten medications prescribed and the top ten diagnoses made are presented in Table 2 and Table 3, respectively.

### 3.2. Multivariable Analysis

The multivariable analysis controlling for disease state, case demographics, health system characteristics, clinical characteristics, and year indicated that DFI cases were independently associated with a significantly increased odds of hospital admission and length of stay across the entire study time frame (*p* < 0.001). Specifically, DFI cases were associated with an odds of admission to the hospital that was 3.002 times higher (CI: 2.145–4.203, *p* < 0.001), and a length of stay that was 1.550 times longer (CI: 1.241–1.936, *p* < 0.001). There were no significant association between DFI cases and 72 h ED revisit (*p* > 0.05). The full multivariable analysis is presented in Table 4.

Beyond DFIs, there were observed associations between the Deyo–Charlson Comorbidity Index and ESI with hospital admission and length of stay in persons with diabetes. Therein, the ESI was associated with increased adjusted admission odds in the worst cases (OR _ESI Level-2_ = 2.326, *p* < 0.001 and OR _ESI Level-1_ = 2.006, *p* < 0.001), and with decreased admissions in less severe, ESI Level-4 cases (OR = 0.146, *p* < 0.001). Furthermore, ESI Level-1 cases were also associated with a 1.242 times longer length of stay than ESI Level-3 or urgent cases (CI: 1.033–1.492, *p* < 0.05). The Deyo–Charlson Comorbidity Index was associated with an odds of hospitalization that was 1.305 times higher (CI: 1.254–1.359, *p* < 0.001) and an inpatient length of stay that was 1.031 times longer (CI: 1.003–1.061, *p* < 0.05).

Regarding demographics, age and male sex were associated with a higher adjusted odds of hospitalization in persons with diabetes (*p* < 0.001). Black race was associated with 22.0% lower adjusted odds of hospitalization compared to white race (*p* < 0.001), while other race was associated with 34.2% higher adjusted odds of admission (*p* < 0.05). Compared to private insurance, cases indicating an expected payment source of a public healthcare coverage were associated with at least one of all three outcomes (i.e., 72 h ED revisit, hospitalization, and length of stay). More specifically, dual coverage (i.e., Medicare plus Medicaid) was significantly associated with an increase in all three outcomes (*p* < 0.05), Medicare coverage was associated with an increase in length of stay (*p* < 0.05), and Medicaid was associated with an increased rate of 72 h ED revisit. Regarding health system characteristics, rural ED locations were associated with lower adjusted hospitalizations and lengths of stay (*p* < 0.05), the Western region was associated with lower hospitalizations relative to the Northeast (*p* < 0.05), and the Midwest was associated with a shorter length of stay (*p* < 0.05).

A sensitivity analysis was conducted to assess if results varied in the COVID-19 pandemic (i.e., ICD-10-CM “U.071”). Therein, results remained consistent in significance and direction concerning DFIs, as no cases of COVID-19 were observed in the DFI cohort. However, a diagnosis of COVID-19 was independently associated with increased odds of hospital admission (OR = 3.016, CI: 2.155–4.221, *p* < 0.001) and an increased length of stay (IR = 1.557, CI: 1.249–1.940, *p* < 0.001), though this was not significantly associated with a 72 h ED revisit (*p* > 0.05). Full results of the COVID-19 era appear in Table 1, Table 2, Table 3 and Table 4.

## 4. Discussion

The current cross-sectional, nationally representative study of 2.4 million DFI-related visits to EDs in the U.S. from 2012 to 2021 observed a 3.002 times higher adjusted odds of hospital admission (CI: 2.145–4.203, *p* < 0.001) and a 55.0% longer length of stay (IR = 1.550, CI: 1.241–1.936, *p* < 0.001) compared to diabetes without foot complications. Six percent of DFI cases involved a 72 h ED revisit, though this was not significantly different than the 4.7% of diabetes (*p* > 0.05). Unadjusted analysis indicated that almost half of DFI cases (43.7%) were admitted to hospital, with an average length of stay of 8.4 ± 5.7 days. Approximately one-third of all DFI cases (31.8%) reported the use of an anti-infective agent with Methicillin-resistant *Staphylococcus aureus* (MRSA) (e.g., vancomycin) or *Pseudomonas aeruginosa* (e.g., piperacillin-tazobactam) coverage. ‘Cellulitis or acute lymphangitis involving an area of the foot’ was the most common diagnosis and was observed in 47.8% of DFI cases. The current study improves upon previous research by including measures of case–mix severity (i.e., Deyo–Charlson Comorbidity Index) and case severity (i.e., ESI) to update national estimates of DFI in emergency care through 2021. The current study also presents medication prescription patterns and top diagnoses involved in DFI and diabetes cases in EDs across this ten-year time horizon.

Previous research on the national trends in diabetic foot complication has largely focused on amputations, though some studies included additional analyses concerning resource utilization [25]. In a study utilizing national ED discharge data from the Agency for Healthcare Research and Quality (AHRQ) Healthcare Cost and Utilization Project (HCUP) Nationwide Emergency Department Sample (NEDS), Skrepnek et al. (2015) reported that one million (1.9%) of all diabetic patients that presented to an ED had a DFU or DFI, and 81.2% of DFU/DFI cases were admitted to hospital from 2006 to 2010 [12]. Following admission, 10.5% underwent amputation, 9.6% had a diagnosis of sepsis, and there was a 2.0% mortality [12]. Patients with Medicaid coverage incurred an adjusted odds of major amputation that was 21% higher compared to those with Medicare (*p* < 0.001), while patients with private insurance, self-pay, and other health coverage were not significantly different than Medicare [12]. A diagnosis of sepsis was present in 9.6% of overall DFU/DFI cases presenting to an ED and was associated with an adjusted odds of minor amputation that was 1.3 times higher (*p* < 0.001), an adjusted odds of major amputation that was 3.9 times higher (*p* < 0.001), and an adjusted odds of inpatient death that was 6.1 times higher [12]. In the current study, 5.7% of DFI cases presented with sepsis, significantly more than the 1.7% in diabetes without foot complications (*p* < 0.001). It is notable that the adjusted odds involving sepsis was not included as an independent factor in the multivariable model due to small sample size of raw observations in DFI cases (i.e., n = 13) [16]. A post hoc sensitivity analysis, however, indicated that including sepsis to the current study’s multivariable model did not change significance of the other variables.

Concerning severe outcomes, Skrepnek et al. (2015) reported that the adjusted odds of major amputation and inpatient death significantly improved over time [12]. Other research has also observed that a decline in diabetes-related amputations occurred from 1990 to 2010 [13,14]. However, Geiss et al. (2018) reported that amputations following this time increased [15]. Though the authors observed a decreased crude rate of diabetes-related, age-adjusted nontraumatic lower-extremity amputations (43%) occurring from 2000 to 2009 (i.e., from 5.38/1000 adults in 2000 to 3.07/1000 in 2009), they also reported a 50% increased rate from 2010 to 2015 (i.e., from 3.07/1000 in 2009 to 4.62/1000 in 2015) [15]. The increased amputation rates observed were particularly in the age ranges of 18 to 44 and 45 to 64 years of age [15]. The rise in diabetes-related amputations in the period from 2010 to 2015 was driven by a 62% increase in the rate of minor amputations (*p* < 0.001) and a 29% increase in major amputations (*p* < 0.001) [15]. The rates of amputations in Geiss et al. (2018) were not adjusted within a multivariable analysis, and no reporting of ulcer, infection, or case–mix severity was included in the study [15].

Concerning the number of preventable hospitalizations, Shrestha et al. (2018) reported that hospitalizations due to lower-extremity amputations and long-term complications of diabetes increased from 2001 to 2014 via HCUP NIS data. While the number of diabetes cases increased, the proportion of lower-extremity amputations and long-term complications decreased [26]. The current study, similarly, indicated an increase in diabetes-related admissions with declining proportion involving DFIs.

In a study that also utilized HCUP NIS data from 2005 to 2010 comparing foot outcomes between DFU and non-diabetes foot ulceration inpatient episodes, Hicks et al. (2016) observed that 58.6% of DFU admissions involved neuropathy and 30.8% involved infection [27]. Infection rates were found to have significantly increased over time for DFU admissions, whereas infection rates remained stable for non-diabetes admissions. The average number of DFU admissions involving infection was approximately 45,000 per year across 2005 to 2010 compared to approximately 5100 for non-diabetes admissions [27]. DFU admissions represented 82.9% of all major amputations from 2005 to 2010, although the overall proportion of DFU admissions requiring a major amputation was significantly lower than that for non-diabetes admissions (2.0%_Diabetes_ vs. 4.6%_Non-diabetes_, *p* < 0.001) [27]. In multivariable analysis controlling for patient- and hospital-level factors, DFU admissions had a 30% significantly lower odds of major amputation compared to non-diabetes (OR 0.7, CI: 0.64–0.77, *p* < 0.001), but with a significant, 3.27-times-higher odds of minor amputation (CI: 3.02–3.55, *p* < 0.001) [27]. Infection was not associated with a difference in major amputation between DFU and non-diabetes admissions but was associated with a 4.58-times-higher odds of minor amputation in DFU versus non-diabetes (CI: 4.10–5.11, *p* < 0.001).

A post hoc analysis of the current study indicated that cellulitis/lymphangitis involving an area of the foot was observed in 47.8% of DFI cases, whereas gangrene was observed in 1.3% of DFI cases. Duhon et al. (2016) analyzed 1.1 million DFI cases across 15 years using the CDC National Hospital Discharge Survey data from 1996 to 2010 [11]. The incidence of DFIs, measured as DFIs over 100 diabetes-related discharges, decreased by half across the study period, from 2.3% in 1996 to 1.1% in 2010 [11]. The most common types of infections reported were gangrene (38.9%), foot cellulitis–abscess (20.7%), osteomyelitis (15%), and toe cellulitis–abscess (7.7%) [11]. Appearing in Table 3 of the current study, cellulitis/lymphangitis was among the top ten diagnoses observed in DFI cases. Post hoc analyses also indicated that a diagnosis of osteomyelitis was observed in 11.8% of DFI cases compared to the 15% reported by Duhon et al. (2016) [11].

### Limitations

Despite the multivariable approach employed, all non-randomized observational studies may inherently be subject to potential selection bias and residual confounding, in part, due to unmeasurable variables. Specifically, NHAMCS data lack laboratory findings (i.e., to assess glycemic control), wound culture findings, and direct assessment of wound/infection severity (e.g., Wagner scale) [28,29]. In the current work, a validated measure for case–mix severity was utilized (i.e., the calculated Deyo–Charlson Comorbidity Index), as was a validated case severity measure (i.e., the ESI) [21,22]. Given inherent data limitations with ICD-10-CM codes within NHAMCS (i.e., truncation of ICD-10-CM codes to 4 digits), the present study utilized a validated algorithm by Sohn et al. (2010) plus the NHAMCS’ reason-for-visit codes to capture DFI cases [16,18,19]. Therein, the relatively consistent number of DFIs across the study time frame, irrespective of changes from ICD-9-CM to ICD-10-CM coding changes, warrants some confidence with the case identification approach used. Beyond the aforementioned, caution is inherently warranted in generalizing findings to specific populations or health systems.

## 5. Conclusions

This nationally representative study of 2.4 million DFI-related ED visits in the U.S. observed a 3.002 times higher odds of hospital admission and 55.0% longer length of stay for DFIs versus diabetes without foot complications. Anti-infective medications were prescribed in an ED in 83.1% of DFI cases, including vancomycin among 28.1%. Further research should seek to assess and develop prevention and coordinated treatment interventions prior to the emergence of DFIs requiring ED care.

## Figures and Tables

**Table 1:** Descriptive statistics of adult emergency department visits related to diabetic foot infections (DFIs) and diabetes without foot complications in the U.S., 2012–2021 (weighted).

	2020–2021			2020–2021 (COVID-19 Era)		
	DFI ^A^	Diabetes	Overall	DFI ^A^	Diabetes	Overall
	*n* = 2,372,132 (estimated)	*n* = 150,596,278 (estimated)	*n* = 1,074,254,782 (estimated)	*n* = 526,948 (estimated)	*n* = 34,685,239 (estimated)	*n* = 219,343,677 (estimated)
Demographics						
Age (mean ± SD)	57.4 ± 12.9 *	60.2 ± 14.4	47.3 ± 17.8	61.2 ± 8.6	61.4 ± 12.5	48.9 ± 15.7
Male (%)	62.1 ***	45.2 ***	43.1	70.2 *	46.1 *	45.0
Race (%)						
White	80.5 **	70.3 **	72.7	82.1	71.9	72.8
Black	16.5 **	25.6 **	23.8	12.0 *	23.6 *	23.1
Other	3.0	4.1	3.5	5.9	4.5	4.2
Ethnicity (%)						
Hispanic	14.1	13.2	13.3	8.9	12.2	13.2
Primary Insurance Coverage (%)						
Private Insurance	23.5	20.6	29.1	23.1	19.6	28.0
Medicare	35.7	38.4	21.6	47.4	40.8	23.7
Medicaid	19.8	21.5	27.3	15.8	23.2	30.4
Dual-Coverage ^B^	7.9	11.3	5.3	8.1	10.1	5.1
Other	13.0	8.3	16.6	5.5	6.3	12.8
Health System Characteristics						
Rural Physician Practice ^C^ (%)	15.6	14.8	14.2	15.9	13.8	12.3
Geographic Region (%)						
Northeast	12.7	15.3	16.8	9.3	16.8	17.4
Midwest	23.1	23.8	22.6	15.0	24.0	21.3
South	39.5	40.2	38.6	51.8	36.1	37.0
West	24.7	20.7	22.0	23.9	23.1	24.3
Clinical Characteristics						
Deyo–Charlson Comorbidity Index (mean ± SD)	1.8 ± 1.1	2.0 ± 1.2	0.6 ± 1.0	2.5 ± 1.2	2.2 ± 1.1	0.7 ± 1.0

Abbreviations: DFI = diabetic foot infection; SD = standard deviation; ED = emergency department. Data are mean ± SD or %. Unweighted sample sizes: DFI _2012 to 2019_ = 343; diabetes without foot complications _2012 to 2019_ = 21,570; overall _2012 to 2019_ = 206,150; DFI _2020 to 2021_ = 52; diabetes without foot complications _2020 to 2021_ = 3844; overall _2020 to 2021_ = 31,067. ^A^ DFI cases determined using ICD-9-CM and ICD-10-CM diagnosis codes and CDC “reason for visit” codes. ^B^ Case indicating both Medicare and Medicaid as expected source of payment. ^C^ Rural practice facility designated as non-metropolitan statistical area. *** *p* < 0.001;** *p* < 0.01;* *p* < 0.05 (independent group *t*-test or χ2 vs. patients with diabetes without foot complications).

**Table 2:** Top 10 all-listed drugs prescribed for adult emergency department visits related to diabetic foot infections (DFIs) and diabetes without foot complications in the U.S., 2012–2021 (weighted).

Diabetic Foot Infection (DFI)			
2012–2021 (estimated *n* = 2,372,132)	%	2020–2021 (estimated *n* = 526,948)	%
1. Vancomycin	28.1	1. Vancomycin	25.6
2. Cephalexin	17.2	2. Piperacillin-Tazobactam	18.0
3. Piperacillin-Tazobactam	15.6	3. Cephalexin	17.1
4. Sulfamethoxazole-Trimethoprim	13.9	4. Acetaminophen	15.4
5. Clindamycin	13.5	5. Clindamycin	15.3
6. Ondansetron	12.0	6. Insulin	14.5
7. Acetaminophen	11.5	7. Ceftriaxone	13.4
8. Hydrocodone-Acetaminophen	11.0	8. Morphine	11.5
9. Insulin	10.9	9. Iopamodil	11.3
10. Morphine	9.2	10. Labetalol	11.3
Diabetes without Foot Complications			
2012–2021 (estimated *n* = 150,596,278)	%	2020–2021 (estimated *n* = 34,685,239)	%
1. Ondansetron	18.2	1. Ondansetron	18.9
2. Acetaminophen	10.4	2. Acetaminophen	15.5
3. Aspirin	8.5	3. Ketorolac	8.3
4. Hydrocodone-Acetaminophen	8.1	4. Aspirin	7.9
5. Morphine	7.9	5. Morphine	7.3
6. Ketorolac	7.5	6. Ceftriaxone	7.2
7. Albuterol	5.4	7. Albuterol	6.2
8. Ceftriaxone	5.4	8. Lidocaine	6.0
9. Insulin	5.2	9. Hydrocodone-Acetaminophen	5.6
10. Hydromorphone	5.1	10. Ibuprofen	5.3

Unweighted sample sizes: DFI _2012–2019_ = 343; diabetes without foot complications _2012 to 2019_ = 21,570; overall _2012 to 2019_ = 206,150; DFI _2020 to 2021_ = 52; diabetes without foot complications _2020 to 2021_ = 3844; overall _2020 to 2021_ = 31,067. Drug identities were determined via Cerner’s Multum^®^ generic identifier.

**Table 3:** Top 10 diagnoses for adult emergency department visits related to diabetic foot infections (DFIs) and diabetes without foot complications in the U.S., 2012–2021 (Weighted).

Diabetic Foot Infection (DFI)			
2012–2021 (estimated *n* = 2,372,132)	%	2020–2021 (estimated *n* = 526,948)	%
1. Cellulitis and acute lymphangitis of other parts of limb	37.3	1. Non-pressure chronic ulcer of other part of foot	30.0
2. Type 2 diabetes mellitus with other specified complications	19.3	2. Type 2 diabetes mellitus with circulatory complications	28.4
3. Non-pressure chronic ulcer of other part of foot	13.4	3. Type 2 diabetes mellitus with other specified complications	28.2
4. Cellulitis and acute lymphangitis of finger and toe	11.1	4. Osteomyelitis, unspecified	13.6
5. Type 2 diabetes mellitus without mention of complications	9.6	5. Cellulitis and acute lymphangitis of other parts of limb	13.0
6. Essential (primary) hypertension	8.7	6. Hyperlipidemia, unspecified	11.9
7. Cellulitis and acute lymphangitis	8.6	7. Atherosclerotic heart disease of native coronary artery	9.6
8. Local infection of the skin and subcutaneous tissue, unspecified	7.7	8. End stage renal disease	9.3
9. Osteomyelitis, unspecified	7.5	9. Cellulitis and acute lymphangitis of finger and toe	8.3
10. Type 2 diabetes mellitus with circulatory complications	7.1	10. Type 2 diabetes mellitus without complications	7.4
Diabetes without Foot Complications			
2012–2021 (estimated *n* = 150,596,278)	%	2020–2021 (estimated *n* = 34,685,239)	%
1. Type 2 diabetes mellitus without complications	15.1	1. Type 2 diabetes mellitus without complications	12.4
2. Essential (primary) hypertension	11.5	2. Essential (primary) hypertension	10.7
3. Chest pain, unspecified	7.3	3. Chest pain, unspecified	7.1
4. Urinary tract infection, site not specified	4.7	4. Acute kidney failure, unspecified	5.1
5. Unspecified abdominal pain	4.3	5. Urinary tract infection, site not specified	4.5
6. Heart failure, unspecified	3.0	6. Dyspnea	4.1
7. Pneumonia, unspecified organism	2.6	7. COVID-19	3.7
8. Acute kidney failure, unspecified	2.4	8. Unspecified abdominal pain	3.6
9. Dyspnea	2.4	9. Hyperglycemia, unspecified	3.4
10. Hyperglycemia, unspecified	2.3	10. Pain in limb, hand, foot, fingers and toes	3.0

Unweighted sample sizes: DFI _2012 to 2019_ = 343; diabetes without foot complications _2012 to 2019_ = 21,570; overall _2012 to 2019_ = 206,150; DFI _2020 to 2021_ = 52; diabetes without foot complications _2020 to 2021_ = 3844; overall _2020 to 2021_ = 31,067. Diagnoses were determined via ICD-9-CM and ICD-10-CM diagnosis codes.

**Table 4:** Multivariable regression analyses of study outcomes of emergency department readmission within 72 h, hospitalization, and length of hospital stay of adult emergency department visits involving diabetic foot infection or diabetes without foot complications in the U.S., 2012–2021 (weighted).

	2012–2021			2012–2021 (COVID-19 Era)		
	Seen in ED in Last 72 h ^A^	Hospitalization ^A^	Length of Stay ^B^	Seen in ED in Last 72 h ^A^	Hospitalization ^A^	Length of Stay ^B^
	[Odds Ratio (95% CI)]	[Odds Ratio (95% CI)]	[Incidence Ratio (95% CI)]	[Odds Ratio (95% CI)]	[Odds Ratio (95% CI)]	[Incidence Ratio (95% CI)]
Disease State						
DFI ^C^	0.813 (0.404,1.634)	3.002 *** (2.145,4.203)	1.550 *** (1.241,1.936)	0.811 (0.404,1.631)	3.016 *** (2.155,4.221)	1.557 *** (1.249,1.940)
Demographics						
Age	0.995 (0.987,1.002)	1.020 *** (1.017,1.024)	1.001 (0.998,1.005)	0.995 (0.987,1.002)	1.020 *** (1.017,1.024)	1.001 (0.998,1.004)
Male	1.129 (0.945,1.349)	1.275 *** (1.161,1.401)	1.072 (0.996,1.154)	1.131 (0.947,1.351)	1.271 *** (1.159,1.395)	1.065 (0.986,1.150)
Race (ref. White)						
Black	0.962 (0.726,1.273)	0.780 *** (0.680,0.894)	1.080 (0.946,1.232)	0.964 (0.728,1.277)	0.776 *** (0.676,0.891)	1.074 (0.942,1.224)
Other	0.997 (0.610,1.628)	1.342 * (1.062,1.695)	1.020 (0.825,1.262)	0.993 (0.608,1.622)	1.354 * (1.072,1.710)	1.031 (0.834,1.273)
Ethnicity						
Hispanic	1.187 (0.684,2.060)	0.870 (0.746,1.015)	0.959 (0.846,1.087)	1.190 (0.686,2.064)	0.865 (0.740,1.012)	0.938 (0.827,1.064)
Primary Insurance Coverage (ref. Private Insurance)						
Medicare	1.300 (0.894,1.890)	1.127 (0.958,1.325)	1.134 * (1.014,1.269)	1.296 (0.892,1.884)	1.133 (0.962,1.334)	1.143 * (1.026,1.273)
Medicaid	1.632 * (1.119,2.379)	0.960 (0.829,1.112)	1.144 (0.980,1.335)	1.628 * (1.116,2.375)	0.965 (0.832,1.119)	1.155 (0.996,1.339)
Dual	1.790 ** (1.185,2.702)	1.199 * (1.013,1.420)	1.271 *** (1.113,1.450)	1.787 ** (1.184,2.699)	1.203 * (1.014,1.426)	1.268 *** (1.114,1.443)
Other	1.516 * (1.011,2.272)	0.826 (0.673,1.015)	1.255 (0.922,1.709)	1.513 * (1.009,2.269)	0.826 (0.673,1.016)	1.264 (0.925,1.728)
Health System Characteristics						
Rural Physician Practice ^D^	1.092 (0.809,1.474)	0.736 * (0.562,0.965)	0.802 ** (0.683,0.943)	1.090 (0.808,1.470)	0.738 * (0.561,0.969)	0.804 ** (0.690,0.937)
Geographic Region (ref. Northeast)						
Midwest	0.830 (0.587,1.174)	0.844 (0.699,1.019)	0.878 * (0.794,0.970)	0.831 (0.588,1.174)	0.842 (0.698,1.016)	0.869 ** (0.788,0.958)
South	1.062 (0.688,1.640)	0.847 (0.687,1.043)	0.948 (0.842,1.068)	1.064 (0.690,1.641)	0.844 (0.686,1.039)	0.942 (0.838,1.059)
West	1.162 (0.800,1.688)	0.759 * (0.607,0.949)	0.972 (0.852,1.109)	1.162 (0.801,1.686)	0.760 * (0.609,0.948)	0.970 (0.849,1.107)
Clinical Characteristics						
Deyo–Charlson Comorbidity Index	0.993 (0.931,1.059)	1.305 *** (1.254,1.359)	1.031 * (1.003,1.061)	0.992 (0.930,1.057)	1.309 *** (1.258,1.362)	1.035 * (1.006,1.065)
Emergency Severity Index						
Level-5 (Best)	0.921 (0.700,1.211)	1.033 (0.909,1.173)	0.948 (0.832,1.081)	0.923 (0.702,1.213)	1.029 (0.907,1.168)	0.947 (0.831,1.078)
Level-4	1.27 (0.847,1.905)	0.146 *** (0.0930,0.231)	0.932 (0.589,1.473)	1.271 (0.847,1.906)	0.146 *** (0.093,0.231)	0.940 (0.594,1.487)
Level-3 ‘Urgent’ (referent)						
Level-2	0.942 (0.733,1.210)	2.326 *** (2.071,2.613)	1.057 (0.925,1.209)	0.952 (0.741,1.223)	2.288 *** (2.037,2.569)	1.045 (0.915,1.192)
Level-1 (Worst)	1.071 (0.702,1.634)	2.006 *** (1.648,2.441)	1.242 * (1.033,1.492)	1.077 (0.705,1.645)	1.997 *** (1.640,2.432)	1.239 * (1.033,1.487)
COVID-19	--	--	--	0.335 * (0.124,0.903)	3.298 *** (1.910,5.692)	1.493 * (1.080,2.064)
Year	0.993 (0.941,1.049)	1.009 (0.983,1.036)	1.010 (0.996,1.025)	0.996 (0.943,1.052)	1.003 (0.977,1.030)	1.006 (0.992,1.021)

Abbreviations: ref. = reference condition; DFI = diabetic foot infection; SD = standard deviation; ED = emergency department; CI = confidence interval. ^A^ Binomial/logistic generalized linear model. ^B^ Negative binomial generalized linear model. ^C^ DFI cases were determined using ICD-9-CM and ICD-10-CM diagnosis codes and CDC “reason for visit” codes. ^D^ Rural practice facility designated as non-metropolitan statistical area. *** *p* < 0.001; ** *p* < 0.01; * *p* < 0.05.

## Data Availability

Publicly available datasets were analyzed in this study and are available with adherence to the Data Use Agreement (DUA) via the CDC’s website for the National Ambulatory Medical Care Survey, Datasets and Documentation. These data may be retrieved at the following: https://ftp.cdc.gov/pub/Health_Statistics/NCHS/Datasets/NHAMCS/ (accessed on 1 February 2024).

## Appendix A

**Table A1:** ICD-9-CM and ICD-10-CM Diagnosis Codes and CDC Reason for Visit Codes.

Diabetes Diagnosis	ICD-9-CM: 250.* ICD-10-CM: E10. *; E11. * CDC priority condition status
Foot-related and non-site-specific infection ^A^	ICD-9-CM: 041.11; 681.1 *; 681.9 *; 682.6 *; 682.7 *; 682.9- 686.9-; 730.06; 730.07; 730.08; 730.09; 730.16; 730.17; 730.18; 730.19; 730.26; 730.27; 730.28; 730.29; 785.4 *; 709.8-; 730.20; 892. *; 893. * ICD-10-CM: A49.9; B95.6; E10.5; E11.5; I96. *; L02.-; L02.6; L02.9; L03.-; L03.0; L03.1; L08.9; L97.-; L97.5; M7.9-; M78.9; M86.1; M86.2; M86.3; M86.4; M86.6; M86.8; M86.9
Reasons for visit (RFV) indicating foot involvement/infection ^A^	1840.3; 1935.0; 2201.0
Reasons for visit (RFV) indicating non-site-specific lesion ^B^	1865.0
Reasons for visit (RFV) indicating foot injury (open wound) ^B^	5030.0; 5545.0
Diagnosis codes potentially including diabetic foot ulcer ^B^	E10.6 ^C^; E11.6 ^C^
Diagnosis code of history of diabetic ulcer ^B^	Z86.3
Reasons for visit (RFV) indicating possible infection ^D^	2035.0; 2800.0; 2910.0

Abbreviations: CDC—Centers for Disease Control and Prevention; * Indicates a wildcard expression; - indicates character not present in diagnosis field. Note: NHAMCS diagnosis codes are truncated to five characters for ICD-9-CM codes and four characters for ICD-10-CM codes; thus, sub-term diagnoses (i.e., longer diagnosis codes) should be assessed for inclusion if these are present. ^A^ A non-site-specific infection diagnosis code required the presence of a foot-specific reason for visit. ^B^ A reason for visit indicating a non-site-specific lesion or an open wound on foot, or a diagnosis code potentially indicating a diabetic foot ulcer required presence of an infection diagnosis (e.g., cellulitis). ^C^ The ICD-10-CM diagnosis codes E10.621 and E11.621 are for diabetic foot ulcer involving Type 1 and Type 2 diabetes, respectively. ^D^ A reason for visit indicating a possible infection (e.g., ‘cellulitis’, ‘osteomyelitis’, ‘staphylococcal infections’) required indication of a wound or ‘other’ culture being ordered.
